# Supplementary material for: Grape Pomace Extracts as Fermentation Medium for the Production of Potential Biopreservation Compounds
Source: Foods. 2019 Feb 2;8(2):51. doi: 10.3390/foods8020051 (PMC6406505; doi:10.3390/foods8020051)
Supplement: Supplementary file 1 [file foods-08-00051-s001.pdf]

Supplementary material

Table S1: Fermentation parameters studied and models used during production of biopreservation compounds by *Candida pyralidae* Y1117, *Pichia kluyveri* Y1125 and *P. kluyveri* Y1164 in a grape pomace extracts medium

Fermentation parameters	Model/Equation	Description
Substrate utilisation rate (g mL^{-1} h^{-1})	$r_s = \frac{dS}{dt}$	This describes the speed of substrate depletion during fermentation. In this case, the depletion rate of total sugar and yeast assimilable nitrogen (YAN) can be considered.
Biomass formation rate (cells mL^{-1} h^{-1})	$r_x = \frac{dX}{dt}$	This describes how fast a specific number of yeast cells are formed during the fermentation period.
Biomass yield (cells g^{-1})	$Y_{X/S} = \frac{dX}{dS}$	This estimates how many cells are formed per gram of the substrate utilised.
Specific growth rate (h^{-1})	$\mu = \frac{ln(X_f/X_0)}{t}$	This quantifies the increase in cell concentration during a specific fermentation period regardless of the availability and preference of the growth controlling substrates.
Biopreservation compounds formation rate (L VZI mL^{-1} BCU h^{-1})	$r_p = \frac{dP}{dt}$	The observed effects of biopreservation compounds are used to assess the level of production during a specific fermentation time.
Biopreservation compound formation based on cell concentration (L VZI cells^{-1})	$Y_{P/X} = \frac{dP}{dX}$	Given the fact that the biopreservation compounds are produced as a result of cellular growth, this model helps to explain and quantify the observed effect of biopreservation compounds in relation to the quantity of cells generated during fermentation.
Biopreservation compounds formation based on substrate utilisation (L VZI g^{-1})	$Y_{P/S} = \frac{dP}{dS}$	Based on how much growth controlling substrate is being utilised during fermentation, this equation correlates the observed effect of biopreservation compounds with the amount of substrate utilised.

Substrate consumption model	$\frac{dS}{dt} = \frac{dX}{Y_{X/S}dt} + \frac{dP}{Y_{P/S}dt} + m_s X$	This model describes the trend of growth controlling substrate utilisation in direct proportion to biomass and product formation, as well as cellular maintenance.

X = Cell concentration (cells mL^{-1}); X_o = Initial cell concentration (cells mL^{-1}); X_f = Final cell concentration (cells mL^{-1}); S = Limiting substrate concentration (g mL^{-1}); P = Biopreservation compounds formation (L VZI mL^{-1} BCU); t = time (h); μ = Specific growth rate (h^{-1}); r_x = Cellular growth rate (cells mL^{-1} h^{-1}); r_P = Volumetric inhibitory activity rate (L CSM mL^{-1} BCU h^{-1}).

Table S2: Process variables, i.e. time, pH, temperature and total sugar used in the central composite design (CCD) for optimisation of biopreservation compounds production by *Candida pyralidae* Y1117, *Pichia kluyveri* Y1125 and *Pichia. kluyveri* Y1164 using grape pomace extracts as fermentation medium

Factors	Units	Code	Low (-1)	High (+1)
Time	H	A	8	40
pH	-	B	2	7
Temperature	°C	C	15	25
Total sugar	g L^{-1}	D	50	180

$Y = \beta_0 + \sum \beta_1 X_1 + \sum \beta_{12} X_1 X_2 + \sum \beta_{11} X_1^2 + \varepsilon$ (Equation 1)

β_1, β_{12} and β_{11}, are the regression coefficients for the linear, interaction and quadratic effects, respectively. The symbols ε, Y and β_0 are random errors, response variables and the intercept value, respectively. The symbols X_1, X_2 (1,2…n) represent the independent variables. Given that 4 independent variables were used in this optimisation study, the equation above then changed to:

$Y = \beta_0 + \beta_1 X_1 + \beta_2 X_2 + \beta_3 X_3 + \beta_4 X_4 + \beta_{12} X_1 X_2 + \beta_{13} X_1 X_3 + \beta_{14} X_1 X_4 + \beta_{23} X_2 X_3 + \beta_{24} X_2 X_4 + \beta_{11} X_1^2 + \beta_{22} X_2^2 + \beta_{33} X_3^2 + \beta_{44} X_4^2 + \varepsilon$ (Equation 2)

Table S3: ANOVA (analysis of variance) for the response surface quadratic model, with A, B, C and D coded for Time (h), pH, Temperature (°C) and Total sugar (g L^{-1}), respectively

Candida pyralidae Y1117

Source	Sum of squares	df	Mean square	F Value	Prob > F	
Model	2.45	14	0.18	200.56	< 0.0001	significant
A-Time	0.049	1	0.049	56.17	< 0.0001	
B-pH	0.12	1	0.12	138.39	< 0.0001	
C-Temp	2.128E-003	1	2.128E-003	2.44	0.1393	
D-Sugar	0.050	1	0.050	57.12	< 0.0001	
AB	4.096E-003	1	4.096E-003	4.69	0.0468	
AC	6.404E-003	1	6.404E-003	7.33	0.0162	
AD	1.894E-004	1	1.894E-004	0.22	0.6481	
BC	0.050	1	0.050	56.98	< 0.0001	
BD	0.039	1	0.039	44.28	< 0.0001	

Pichia kluyveri Y1125

**Source**	Sum of Squares	df	Mean Square	F Value	Prob > F	
**Model**	0.71	14	0.051	96.05	< 0.0001	significant
**A-Time**	0.015	1	0.015	27.63	< 0.0001	
**B-pH**	0.042	1	0.042	80.03	< 0.0001	
**C-Temp**	5.517E-003	1	5.517E-003	10.39	0.0057	
**D-Sugar**	0.024	1	0.024	46.02	< 0.0001	
**AB**	9.417E-003	1	9.417E-003	17.73	0.0008	
**AC**	8.698E-003	1	8.698E-003	16.38	0.0011	
**AD**	0.014	1	0.014	25.93	0.0001	
**BC**	0.019	1	0.019	36.36	< 0.0001	
**BD**	8.374E-004	1	8.374E-004	1.58	0.2284	

Pichia kluyveri Y1164

Source	Sum of Squares	df	Mean Square	F Value
**Model**	0.99	14	0.071	736.89
**A-Time**	0.027	1	0.027	279.68
**B-pH**	0.22	1	0.22	2255.21
**C-Temp**	0.013	1	0.013	134.91
**D-Sugar**	0.021	1	0.021	214.31
**AB**	0.019	1	0.019	203.25
**AC**	5.156E-003	1	5.156E-003	53.88
**AD**	1.668E-003	1	1.668E-003	17.43
**BC**	5.314E-003	1	5.314E-003	55.52
**BD**	0.012	1	0.012	121.08

CD	0.021	1	0.021	24.40	0.0002	**CD**	0.011	1	0.011	21.50	0.0003	**CD**	1.038E-003	1	1.038E-003	10.85
A2	0.012	1	0.012	13.89	0.0020	**A2**	9.211E-003	1	9.211E-003	17.35	0.0008	**A2**	0.052	1	0.052	539.54
B2	0.035	1	0.035	39.89	< 0.0001	**B2**	1.279E-003	1	1.279E-003	2.41	0.1416	**B2**	0.027	1	0.027	279.59
C2	0.071	1	0.071	81.05	< 0.0001	**C2**	6.142E-003	1	6.142E-003	11.57	0.0040	**C2**	1.315E-003	1	1.315E-003	13.74
D2	3.287E-003	1	3.287E-003	3.76	0.0714	**D2**	0.042	1	0.042	78.58	< 0.0001	**D2**	1.642E-005	1	1.642E-005	0.17
Residual	0.013	15	8.733E-004			**Residual**	7.966E-003	15	5.311E-004			**Residual**	1.436E-003	15	9.570E-005	
Lack of Fit	0.013	10	1.310E-003			**Lack of Fit**	7.966E-003	11	7.242E-004			**Lack of Fit**	1.436E-003	12	1.196E-004	
Pure Error	0.000	5	0.000			**Pure Error**	0.000	4	0.000			**Pure Error**	0.000	3	0.000	
Cor Total	2.47	29				**Cor Total**	0.72	29				**Cor Total**	0.99	29		
Std. Dev.	0.030		R-Squared	0.9947		**Std. Dev.**	0.023		R-Squared	0.9890		**Std. Dev.**	9.783E-003		R-Squared	0.9985
Mean	0.80		Adj R-Squared	0.9897		**Mean**	0.36		Adj R-Squared	0.9787		**Mean**	0.32		Adj R-Squared	0.9972
C.V. %	3.69		Pred R-Squared	0.9721		C.V. %	6.32		Pred R-Squared	0.8385		C.V. %	3.05		Pred R-Squared	0.9927

PRESS	0.069	Adeq Precision	51.553		PRESS	0.12	Adeq Precision	37.755	PRESS	7.232E-003	Adeq Precision	95.431

Table S4: Predicted and observed experimental productivity (L VZI mL^{-1} BCU) runs generated for *Candida pyralidae* Y1117 (a), *Pichia kluyveri* Y1125 (b) and *P. kluyveri* Y1164 (c) by the central composite design (CCD)

***Candida pyralidae* Y1117**

Run	Factors				(L VZI/mL BCU)	
	A (h)	B	C (oC)	D g/L	Predicted	Actual
1	8	4,5	20	5	0,165	0,166
2	12	7	20	11,25	0,496	0,497
3	28	4,5	20	11,25	0,715	0,715
4	28	4,5	20	11,25	0,715	0,715
5	28	3	15	7,5	0,995	1,005
6	16	3	25	7,5	1,017	1,005
7	36	3	15	7,5	0,907	0,913
8	28	4,5	20	11,25	0,715	0,715
9	28	3	25	7,5	1,242	1,237
10	36	6	15	7,5	0,655	0,689
11	24	3	25	15	1,238	1,272
12	32	3	15	7,5	0,964	0,913
13	40	6	25	7,5	0,191	0,192
14	28	6	15	7,5	0,778	0,769
15	24	6	15	15	1,033	1,036
16	28	4,5	20	11,25	0,715	0,715
17	20	3	25	7,5	1,118	1,134
18	8	4,5	20	18	0,413	0,412
19	28	6	15	15	1,007	1,005
20	20	6	15	7,5	0,795	0,769
21	20	3	25	15	1,166	1,168
22	24	2	20	11,25	1,067	1,069
23	36	7	20	11,25	0,561	0,542

***Pichia kluyveri* Y1125**

Run	Factors				(L VZI/mL BCU)	
	A (h)	B	C (oC)	D g/L	Predicted	Actual
1	8	6	15	15	0,147	0,153
2	8	4,5	20	5	0,140	0,119
3	12	3	15	7,5	0,262	0,284
4	12	4,5	20	18	0,213	0,206
5	12	4,5	20	5	0,172	0,166
6	16	4,5	20	5	0,194	0,206
7	20	6	15	15	0,315	0,336
8	20	7	20	11,25	0,316	0,301
9	24	4,5	20	11,25	0,432	0,433
10	24	4,5	20	11,25	0,432	0,433
11	24	4,5	20	11,25	0,432	0,433
12	24	4,5	20	11,25	0,432	0,433
13	24	6	15	15	0,350	0,336
14	24	4,5	20	11,25	0,432	0,433
15	24	6	15	7,5	0,365	0,354
16	24	6	25	7,5	0,229	0,251
17	24	7	20	11,25	0,304	0,318
18	24	3	15	15	0,489	0,475
19	28	4,5	20	18	0,380	0,412
20	28	3	25	15	0,715	0,742
21	28	6	25	15	0,358	0,336
22	28	7	20	11,25	0,281	0,284
23	28	3	15	15	0,549	0,519

***Pichia kluyveri* Y1164**

Run	Factors				(L VZI/mL BCU)	
	A (h)	B	C (oC)	D g/L	Predicted	Actual
1	8	4.5	20	11.25	0,130	0.12977
2	8	4.5	20	11.25	0,130	0.12977
3	8	4.5	20	11.25	0,130	0.12977
4	8	4.5	20	11.25	0,130	0.12977
5	8	4.5	20	5	0,096	0.098125
6	8	6	25	7.5	0,175	0.178833
7	8	7	20	11.25	0,171	0.165831
8	12	3	25	15	0,438	0.432731
9	12	6	15	7.5	0,178	0.178833
10	12	6	25	15	0,196	0.206308
11	16	6	25	7.5	0,267	0.2512
12	20	6	15	15	0,289	0.300508
13	20	6	25	15	0,267	0.267145
14	20	3	25	7.5	0,450	0.453583
15	20	7	20	11.25	0,297	0.300508
16	24	6	15	15	0,313	0.300508
17	24	6	25	7.5	0,274	0.283581
18	24	3	15	15	0,726	0.715331
19	28	3	15	7.5	0,589	0.588995
20	28	7	20	11.25	0,275	0.267145
21	28	3	15	15	0,756	0.7693
22	32	4.5	20	18	0,409	0.41237
23	32	3	25	15	0,591	0.588995

24	28	4,5	20	11,25	0,715	0,715
25	12	3	25	15	0,945	0,974
26	28	4,5	20	11,25	0,715	0,715
27	24	3	15	7,5	1,000	1,036
28	16	3	25	15	1,069	1,005
29	16	7	20	11,25	0,572	0,589
30	8	3	15	15	0,337	0,336

24	32	4,5	20	18	0,395	0,393
25	32	6	15	15	0,388	0,373
26	32	3	25	15	0,689	0,689
27	36	6	15	15	0,391	0,393
28	36	6	15	7,5	0,344	0,354
29	40	4,5	20	5	0,100	0,089
30	40	3	15	15	0,667	0,689

24	32	6	25	15	0,214	0.206308
25	36	4.5	20	18	0,378	0.37312
26	36	3	25	15	0,558	0.5652
27	36	6	25	15	0,154	0.15332
28	36	7	20	11.25	0,169	0.178833
29	40	3	15	7.5	0,522	0.519081
30	40	3	25	7.5	0,339	0.335833

Table S5: Criteria for the selection of optimum conditions for production of biopreservation compounds (desirability response) in grape pomace extracts as fermentation medium.

Factors	Goal	Lower limit	Upper limit	Lower weight	Upper weight	Importance
Time (h)	In range	4	32	1	1	3
pH	In range	2	7	1	1	3
Temperature (^oC)	In range	15	25	1	1	3
Sugar concentration (g L^{-1})	In range	5	18	1	1	3
Response (L VZI mL^{-1} BCU h^{-1})	Maximise	0.1658	1.2717	1	1	5

Towards constructing the indices of desirability, five goal possibilities were used and the indices used were none, maximum, minimum, target and within range. The criteria for the selection of the optimum conditions for production of biopreservation compounds are shown above and the "importance" value of 5 was considered as the maximum desirable goal. The "importance" value assigned to a parameter shows the level of importance or weight that a specific parameter carries towards achieving the set target.

***Candida pyralidae* Y1117** | ***Pichia kluyveri* Y1125** | ***Pichia kluyveri* Y1164**

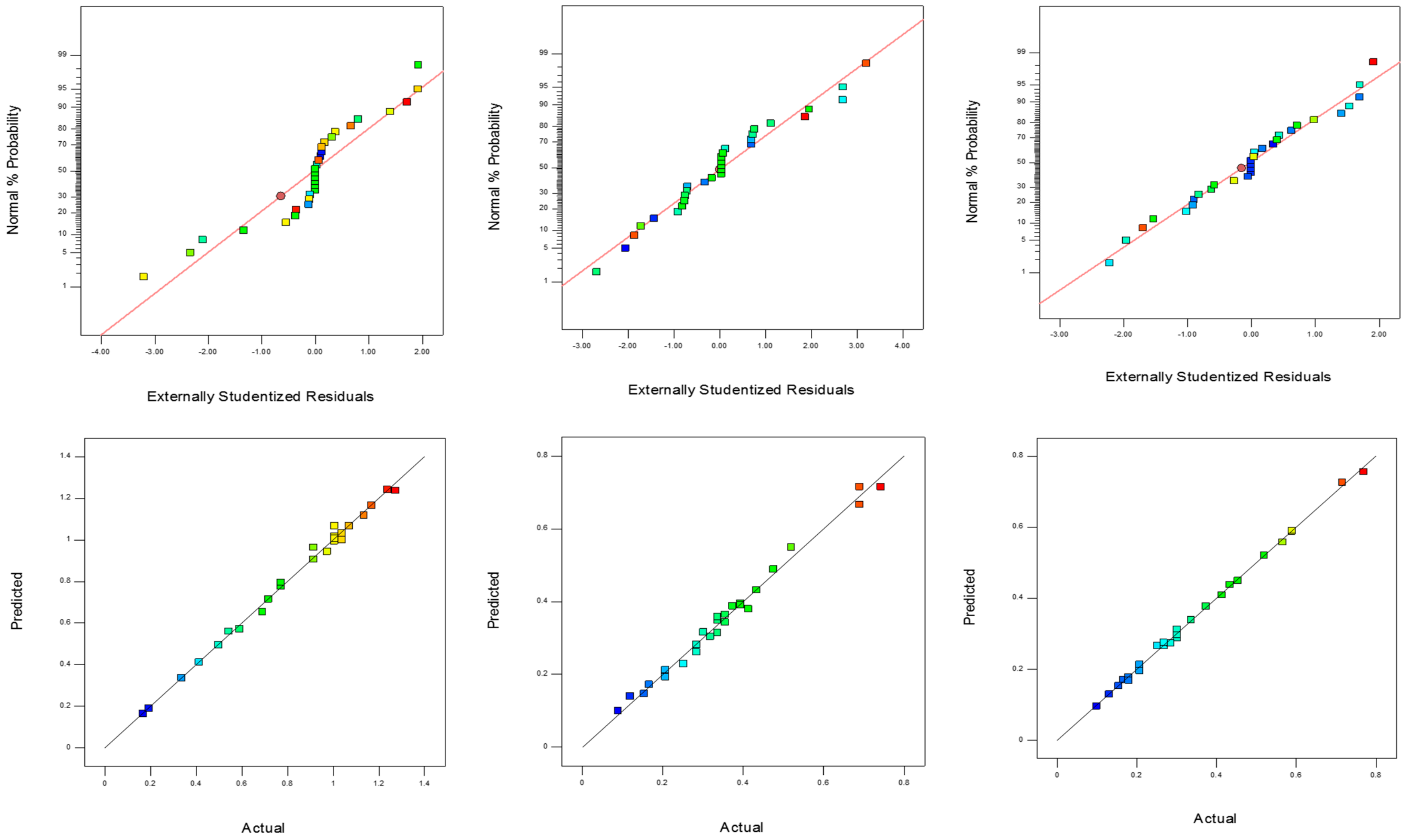

Figure S1: The external studentized residuals versus the normal percentage probability and the actual versus the predicted response plots for production of biopreservation compounds by *Candida pyralidae* Y1117, *Pichia kluyveri* Y1125 *and P. kluyveri* Y1164 in a grape pomace extracts medium

a

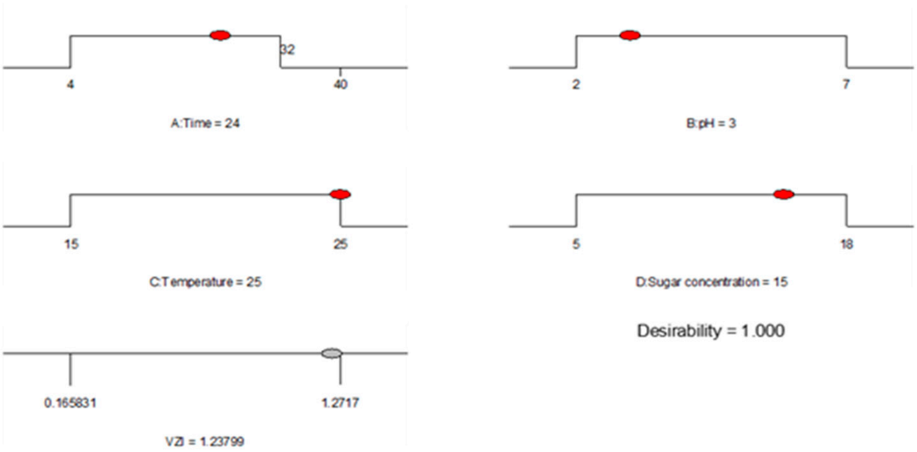
4
32
40
A:Time = 24
2
7
B:pH = 3
15
25
C:Temperature = 25
5
18
D:Sugar concentration = 15
Desirability = 1.000
0.165831
1.2717
VZI = 1.23799

b

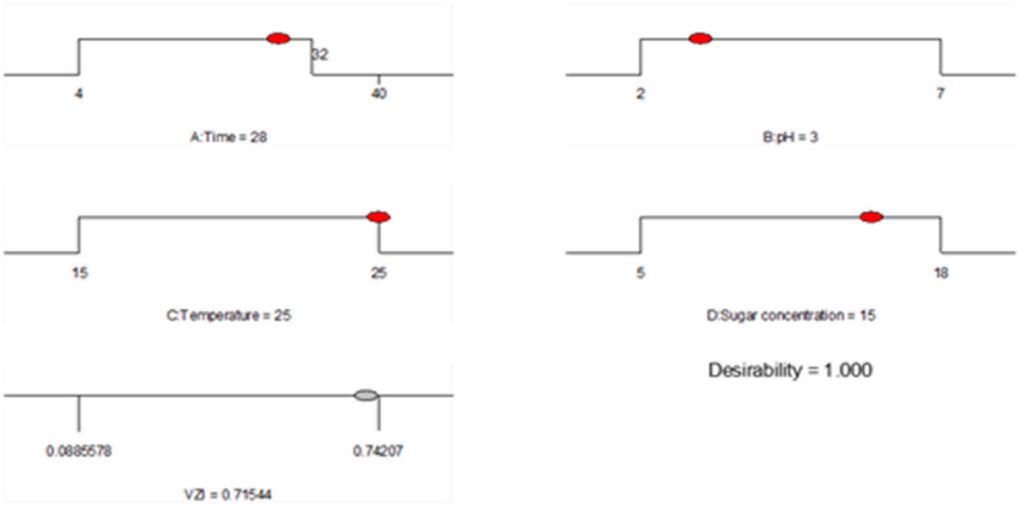
4
32
40
A:Time = 28
2
7
B:pH = 3
15
25
C:Temperature = 25
5
18
D:Sugar concentration = 15
Desirability = 1.000
0.0885578
0.74207
VZI = 0.71544

c

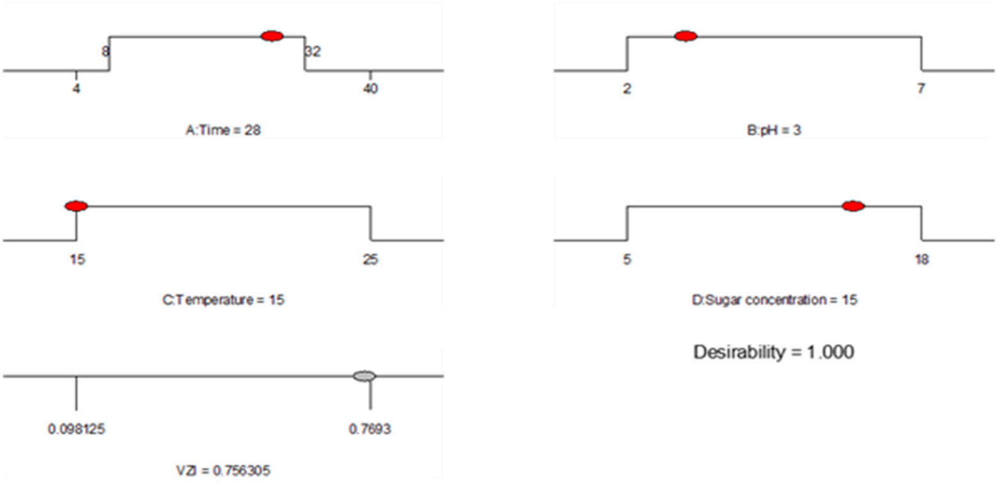
4
8
32
40
A:Time = 28
2
7
B:pH = 3
15
25
C:Temperature = 15
5
18
D:Sugar concentration = 15
Desirability = 1.000
0.098125
0.7693
VZI = 0.756305

Figure S2: Ramp diagram and desirability values for optimal conditions for biopreservation compounds production under the conditions studied (time, pH, temperature and sugar concentration). a, b, c = optimal production conditions for *Candida pyralidae* Y1117, *Pichia kluyveri* Y1125 and *P. kluyveri* Y1164, respectively
